# Agreements to Surrender Practice

**Published:** 1916-09

**Authors:** Arthur L. H. Street

**Affiliations:** St. Paul, Minn.


### AGREEMENTS TO SURRENDER PRACTICE.

BY ARTHUR L. H. STREET, ST. PAUL, MINN.

An instructive court decision bearing upon the validity of an agreement by a dentist in selling his business in a town, not to re-engage in the practice there in competition with his successor is to be found in the case of Murdock v. Locke, 151 *Pacific Reporter,* 298, lately passed upon by the New Mexico Supreme Court.

Plaintiff, a dental surgeon, went from Missouri to New Mexico in response to an advertisement in a dental magazine stating that defendant's business in a town in the latter state was for sale. As a culmination of written and verbal negotiations, plaintiff bought the business and defendant agreed not to open an office in the town for five years for the purpose of practising dentistry therein. Claiming that defendant had violated this agreement, plaintiff brought suit to enjoin further violation of the contract and to recover damages sustained through the breach. The trial court awarded judgment in plaintiff's favor, but on appeal the judgment was reversed so far as it awarded damages, on the ground that plaintiff had failed to establish the amount of his loss. The judgment was affirmed, however, so far as it enjoined further violation of the contract. Here are the principal points decided by the higher court, and it would seem that they fairly state the law applicable to such contracts in all states:

Where two persons enter into a written contract, neither will be permitted to afterwards assert a verbal contract which contradicts the written agreement. All prior and contemporaneous verbal understandings will be presumed to have been merged into the written contract finally signed. But proof may be given of an oral agreement not inconsistent with the written contract, although it may have been made at the same time or before the writing was executed. Thus, it may be shown that when a written contract to sell a dental business was entered into, it was verbally agreed that the seller would refrain from practice in the same town.

Agreement by the seller that he will not re-engage in the practice of dentistry in the same town for a stated period prevents him from opening an office in that town for the purpose of treating persons living outside the town.

The courts will grant injunction against violation of such an agreement.—*Dental Digest.*

